# Regulation of Flowering Timing by ABA-NnSnRK1 Signaling Pathway in Lotus

**DOI:** 10.3390/ijms22083932

**Published:** 2021-04-10

**Authors:** Jing Cao, Qijiang Jin, Jiaying Kuang, Yanjie Wang, Yingchun Xu

**Affiliations:** College of Horticulture, Nanjing Agricultural University, Nanjing 210095, China; 2018104114@njau.edu.cn (J.C.); 15070521551@163.com (J.K.); zjjwyj@njau.edu.cn (Y.W.)

**Keywords:** PCD, ABA, NnSnRK1, flower bud abortion, *Nelumbo nucifera*

## Abstract

The lotus produces flower buds at each node, yet most of them are aborted because of unfavorable environmental changes and the mechanism remains unclear. In this work, we proposed a potential novel pathway for ABA-mediated flower timing control in the lotus, which was explored by combining molecular, genetic, transcriptomic, biochemical, and pharmacologic approaches. We found that the aborting flower buds experienced extensive programmed cell death (PCD). The hormonal changes between the normal and aborting flower buds were dominated by abscisic acid (ABA). Seedlings treated with increasing concentrations of ABA exhibited a differential alleviating effect on flower bud abortion, with a maximal response at 80 μM. Transcriptome analysis further confirmed the changes of ABA content and the occurrence of PCD, and indicated the importance of PCD-related SNF1-related protein kinase 1 (NnSnRK1). The NnSnRK1-silenced lotus seedlings showed stronger flowering ability, with their flower:leaf ratio increased by 40%. When seedlings were treated with ABA, the expression level and protein kinase activity of NnSnRK1 significantly decreased. The phenotype of NnSnRK1-silenced seedlings could also be enhanced by ABA treatment and reversed by tungstate treatment. These results suggested that the decline of ABA content in lotus flower buds released its repression of NnSnRK1, which then initiated flower bud abortion.

## 1. Introduction

The appropriate flowering time is crucial to ensure the reproductive success of plants. Plants demonstrate sophisticated mechanisms to integrate diverse environmental cues and endogenous signals to ensure survival and reproductive success [1]. There has been substantial previous research on the transition from vegetative to reproductive development being controlled by temperature, photoperiod, hormonal status, and available nutrients [2]. Plant hormone signaling pathways are critical hubs that finely adjust growth and reproduction according to different environmental stimuli derived from growth conditions, nutrient availability, and biotic and abiotic stress [3]. Abscisic acid (ABA) is long known to play a central role in the regulation of plant stress responses and is considered to be the stress hormone [4]. However, there is accumulating evidence that ABA is involved in the regulation of reproductive processes [5]. In Arabidopsis, ABA has been revealed as a flowering activator, especially in the drought-escape response [6]. A water-deficit can induce the accumulation of ABA, which regulates many flowering-related genes to promote early flowering in rice [7]. In the presence of ABA, SNF1-related protein kinase 2 (SnRK2) can phosphorylate its downstream targets, including several ABA-responsive transcription factors (ABFs) that mediate the effect of ABA signaling on flowering [8]. Although progress has been made in the ABA-SnRK1 signaling pathway, the role played by it in regulating flowering regulation remains largely unknown.

Sugar metabolism and the partitioning of sugars between source and sink tissues are intimately associated with the time of flowering transition in plants. Accumulating evidence suggest the functional link between flowering time, sugar levels, and sucrose nonfermenting-1 (SNF1)-related protein kinases 1 (SnRK1) activity. SnRK1 has been demonstrated to be an important regulator in the regulation of metabolism and the energy balance of cells [9]. Overexpression of the SnRK1 gene in Arabidopsis delays its flowering time, such as when a mutant of its negative regulator trehalose-6-P synthase 1 (TPS1) flowers much later [1]. INDETERMINATE DOMAIN (IDD8) transcription factor is identified as a downstream target of SnRK1, which regulates flowering time by modulating sugar metabolism, sensing, and transport [10]. Moreover, a high-throughput yeast two-hybrid screen showed 125 ABA-regulated proteins can interact with SnRK1, suggesting a widespread linkage between SnRK1 and ABA signaling pathways.

In contrast to the detailed understanding of flowering transition in plants, little is known about the developmental control of flower buds in response to environmental changes. *Nelumbo nucifera* is an ornamental and edible plant that is widely cultivated. During the growing season, the lotus produces flower buds at each node and most of them are aborted because of unfavorable environmental changes, such as leaf shading by other leaves and rainy weather. Adverse environmental conditions reduce reproductive success, slow seed and fruit development, and even threaten survival. This different strategy could help the lotus to terminate flowering with low reproductive success and minimize energy waste, yet the mechanism remains unclear. In this work, we conducted an integrated analysis of morphological and cytological observations, endogenous phytohormone assay, high throughput sequencing, and transgenic validations to uncover the underlying regulatory mechanism.

## 2. Results and Discussions

### 2.1. Most Lotus Flower Buds Aborted in Unfavorable Environments

**Figure 1:**
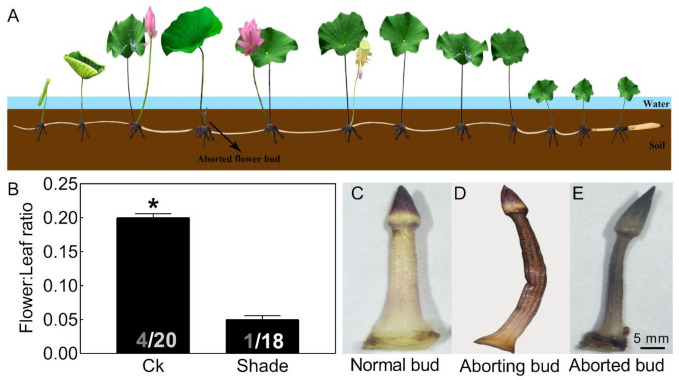
Phenotypic characteristics of lotus flower buds. Two-month old lotus seedlings were used in shading treatment and flower bud observation. (**A**) Schematic representation of lotus growth. (**B**) Flower:leaf ratio of a lotus with or without 50% shading treatment for 6 weeks. The numbers on the bottom of each column represent the corresponding number of flowers and leaves of flower:leaf ratio. (**C**–**E**) Photographs of normal (**C**), aborting (**D**), and aborted lotus flower buds (**E**). Values are the means ± SE of three independent experiments with at least three replicates for each. Bars with asterisks were significantly different in comparison with Ck (no treated) at * *p* < 0.05, according to *t*-test.

Proper flowering timing determines the reproductive success of plants [1]. The lotus (*Nelumbo nucifera*) produces flower buds at each node and most of them are aborted (Figure 1A), yet the mechanism remains unclear [11]. Adverse environmental changes that limit photosynthesis and alter carbohydrate levels often lead to aborted flower buds, i.e., leaf shading by other leaves and rainy weather (Figure 1B), which led to a flower bud abortion rate over 80% in the variety used in this study. Flower bud abortion also exists in other plants, such as the tree peony [12] and rose [13]. In roses, abortion in winter is attributed to a disequilibrium between carbohydrate production and demand [13]. To maximize reproductive success, plants tend to keep the flowering process in coincidence with the most favorable environmental conditions.

Abortion in visible lotus flower buds (about 2 cm) could be divided into three stages. The normal flower bud appeared pink-white (Figure 1C). As the bud abortion proceeded, it firstly turned brown, then gradually lost water and shriveled (Figure 1D). Finally, the flower bud appeared dark brown and aborted (Figure 1E). In our follow-up study, the aborting flower buds were used for further analysis.

### 2.2. Programmed Cell Death (PCD) Might Lead to Flower Bud Abortion in the Lotus 

**Figure 2:**
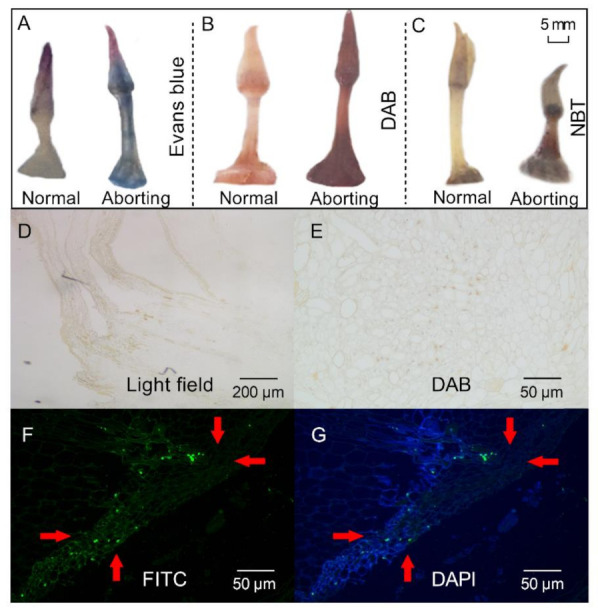
Histochemical staining and TdT-mediated dUTP-biotin nick end labeling (TUNEL) staining of lotus flower buds. After growing for 2 months, 2- to 3-cm-long flower buds were excised for different staining. (**A**–**C**) Evans blue (**A**), 3, 3-diaminobenzidine (DAB) (**B**), and nitro blue tetrazolium chloride (NBT; (**C**)), staining of the normal and aborting flower buds. Bar, 5 mm; (**D**) Bright field view. (**E**–**G**) TUNEL analysis of the aborting flower buds stained with DAB (**E**), fluorescein isothiocyanate (FITC; (**F**)), and 6-diamidino-2-phenylindole (DAPI; (**G**)). The red arrows with the same direction point to the same position on the bud.

Histochemical stainings were performed to analyze the physicochemical state of the aborting flower buds. Staining of the aborting flower buds with Evans blue revealed an intensity of blue staining, indicating that cell death had occurred (Figure 2A). Accumulation of reactive oxygen species (ROS), as shown by 3, 3-diaminobenzidine (DAB; Figure 2B) and nitro blue tetrazolium chloride (NBT) staining (Figure 2C), was observed in the aborting flower buds. ROS production is often related to programmed cell death [14], which is a controlled cell suicide that determines the growth and development of plants [15]. TdT-mediated-dUTP-biotin nick end labeling (TUNEL) analysis was then performed to determine the presence of PCD in the aborting buds [15]. When using routine DAB staining, the nuclei were stained brown in the aborting flower buds, which represented a TUNEL-positive reaction and cells having nuclear changes associated with PCD (Figure 2E). TUNEL-positive cells were also detected by staining with fluorescein isothiocyanate (FITC; Figure 2F). Representative photographs of the tests with FITC staining (green) and counterstained with 4′,6-diamidino-2-phenylindole (DAPI, blue; Figure 2G) showed that the cells in the aborting flower buds were undergoing PCD. These results indicated that PCD might be a key reason for the abortion of lotus flower buds, in line with the notion that PCD is a normal component of flower development [16].

### 2.3. ABA Plays an Important Role in Regulating Lotus Flower Bud Abortion 

**Figure 3:**
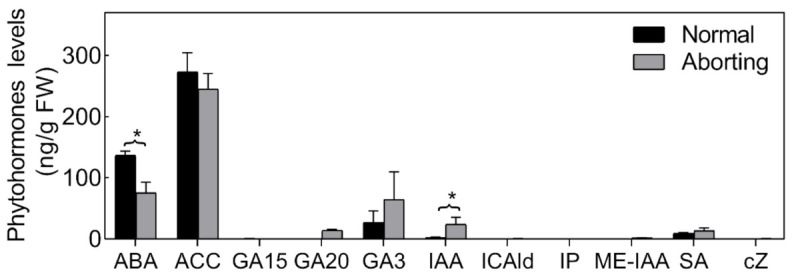
Endogenous hormone proles of normal and aborting flower buds. After growing for 2 months, 2- to 3-m long normal or aborting flower buds were excised for phytohormone or biosynthetic intermediates content analysis. Content of abscisic acid (ABA), 1-Aminocyclopropanecarboxylic acid (ACC), salicylic acid (SA), gibberellin A15 (GA15), GA20, GA3, auxin, indole-3-acetic acid (IAA), methyl indole-3-acetate (ME-IAA), Indole-3-carboxaldehyde (ICAld), N6-isopentenyladenine (IP), and cis-zeatin (cZ) were analyzed. Values are the means ± SE of three independent experiments with at least three replicates for each. Bars with asterisks were significantly different in comparison with normal buds at * *p* < 0.05, according to the *t*-test.

Phytohormones are thought to play important roles in the PCD process [17]. We therefore measured the levels of different bioactive forms or biosynthetic intermediates of cytokinin (CTK), salicylic acid (SA), auxin (IAA), ethylene (Eth), abscisic acid (ABA) and gibberellin (GAs) in both normal and aborting flower buds (Figure 3). The hormonal changes were dominated by significant changes in the level of ABA and IAA. The ABA level was relatively high (>36.67 ng/g fresh weight (FW)) in the flower buds and declined almost 50% in the aborting flower buds. Accordingly, we speculated that exogenous ABA could reduce the abortion rate of flower buds. 

**Figure 4:**
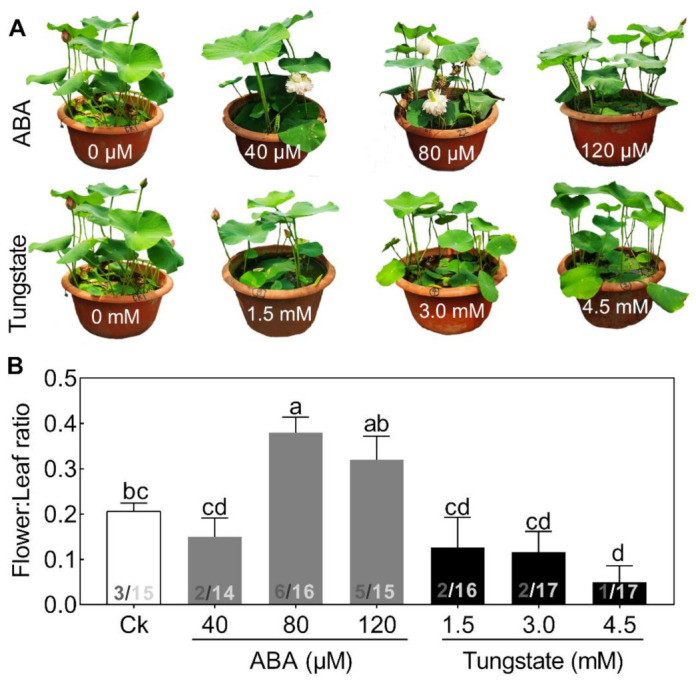
Effects of different concentrations of abscisic acid (ABA) and tungstate on lotus plants. Two-month-old lotus seedlings were used for ABA and tungstate treatments. The representative picture was taken when plants were treated for 9 days (**A**) and the flower:leaf ratio (**B**) was analyzed. The numbers on the bottom of each column represent the corresponding number of flowers and leaves of the flower:leaf ratio. Values are the means ± SE of three independent experiments with at least three replicates for each.Bars marked with different letters are significantly different from each oher at *p* < 0.05, according to Tukey’s multiple test.

To test this hypothesis, exogenous ABA, ranging from 40 to 120 μM, and its inhibitor, tungstate, ranging from 1.5 to 4.5 mM, were added (Figure 4). Seedlings treated with increasing concentrations of ABA exhibited a differential alleviating effect on flower bud abortion, with a maximal response at 80 μM (Figure 4). By contrast, the inhibitor exerted the opposite effect on flower:leaf ratio (Figure 4). The promoting effects of exogenous ABA on flowering have also been reported in several other plant species [5]. These results demonstrated that ABA might play a significant role in regulating lotus flower bud abortion. However, compared with the extensive studies of ABA in plant stress responses, its role in the flowering regulatory network is just beginning to emerge.

### 2.4. Transcriptome Analysis Confirmed Changes in ABA and the Occurrence of PCD

**Figure 5:**
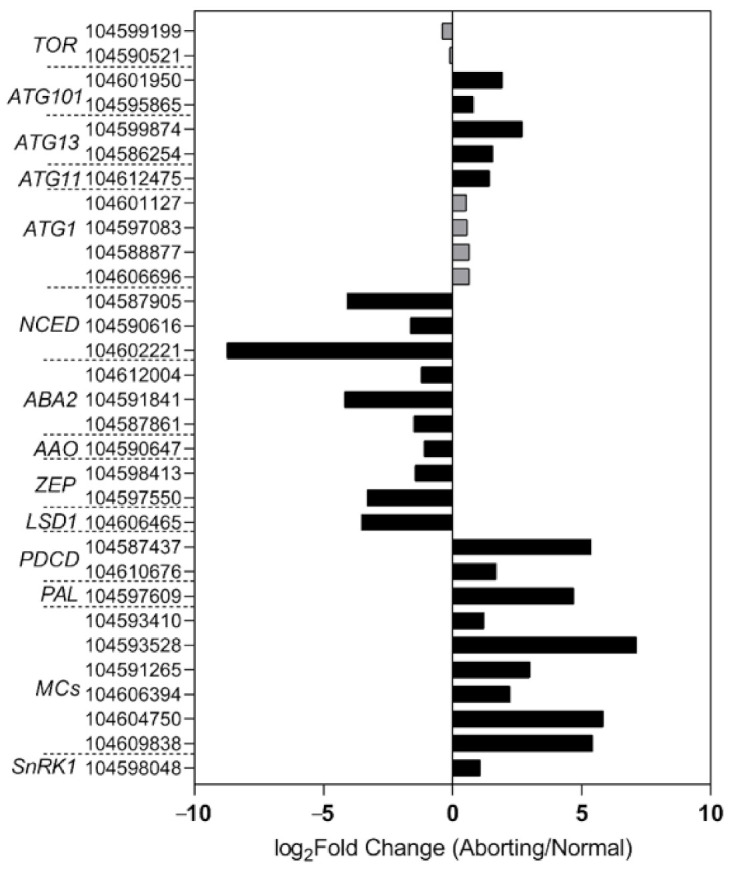
High-throughput sequencing analysis of the normal and aborting lotus flower buds. After growing for 2 months, 2- to 3 -cm-long normal or aborting flower buds were excised for high-throughput sequencing analysis. Black columns indicate significantly changed genes (fold change ≥ 2, adjust *p* value ≤ 0.001) between the normal and aborting flower buds. The numbers are the NCBI accession numbers of corresponding genes. Color scale indicates the expression level of genes in log_2_(aborting/normal)-transformed value.

To gain a better understanding of the regulatory mechanisms in lotus flower bud abortions, we performed RNA-seq analysis of the normal and aborting flower buds. The transcriptomic data showed that key genes involved in the biosynthesis pathway of ABA changed in accordance with the levels of ABA (Figure 5), including *9-cis-epoxycarotenoid dioxygenase* (*NCED*), *xanthoxin dehydrogenase* (*ABA2*), *abscisic-aldehyde oxidase* (*AAO*), and *zeaxanthin epoxidase* [18,19,20]. These results demonstrated that the decrease in ABA content was caused by decreased biosynthesis. Moreover, the expression of genes related to PCD regulation showed significant changes between the normal and aborting buds (Figure 5). Well-known positive regulators, like *programmed cell death protein 4* (*PDCD4*), *PDCD5*, *phenylalanine ammonia-lyase* (*PAL*), and *metacaspase* (*MCs*), and negative regulators, such as *lesion simulating disease 1* (*LSD1*) [17,21], changed significantly in the aborting flower buds (Figure 5).

SNF1-related protein kinase 1 (SnRK1) is supposed to be a key energy sensor in coordinating cell growth with energy availability and a key regulator in ABA signaling [22,23]. Some evidence suggests that SnRK1 is an inducer of PCD [24]. Consistent with this, our transcriptome data showed that *NnSnRK1* accumulated over 1 time higher in the aborting flower buds compared with the normal buds and showed an opposite change compared with ABA levels (Figure 5). This result suggested that NnSnRK1 likely played important roles in flower bud abortion and ABA signaling.

### 2.5. Silencing NnSnRK1 Could Enhance the Flowering Ability of Lotus

**Figure 6:**
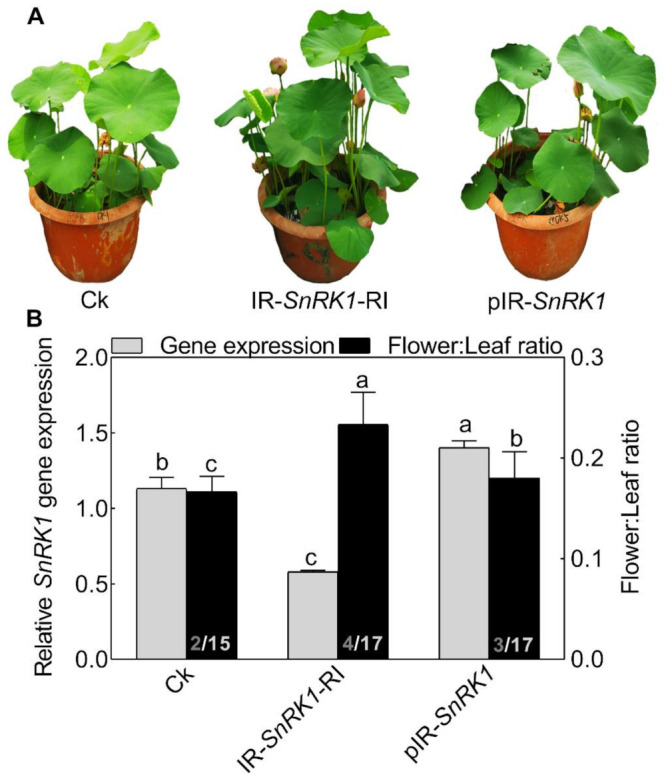
Phenotypes of *NnSnRK1*-silenced (IR-*NnSnRK1*-RI) and -overexpressed (pIR-*NnSnRK1*) lotus. Two-month-old lotus seedlings were used for infection with IL-60-BS-derived systems. IR-*NnSnRK1*-RI, *NnSnRK1*-silenced lotus; pIR-*NnSnRK1*, *NnSnRK1*-overexpressed lotus. The representative picture was taken after infection for 14 days (**A**), and then the gene expression level of *NnSnRK1* genes and the flower:leaf ratio were analyzed (**B**). The numbers on the bottom of each column represent the corresponding number of flowers and leaves of the flower:leaf ratio. Values are the means ± SE of three independent experiments with at least three replicates for each. Bars marked with different letters are significantly different from each other at *p* < 0.05, according to Tukey’s multiple test.

**Figure 7:**
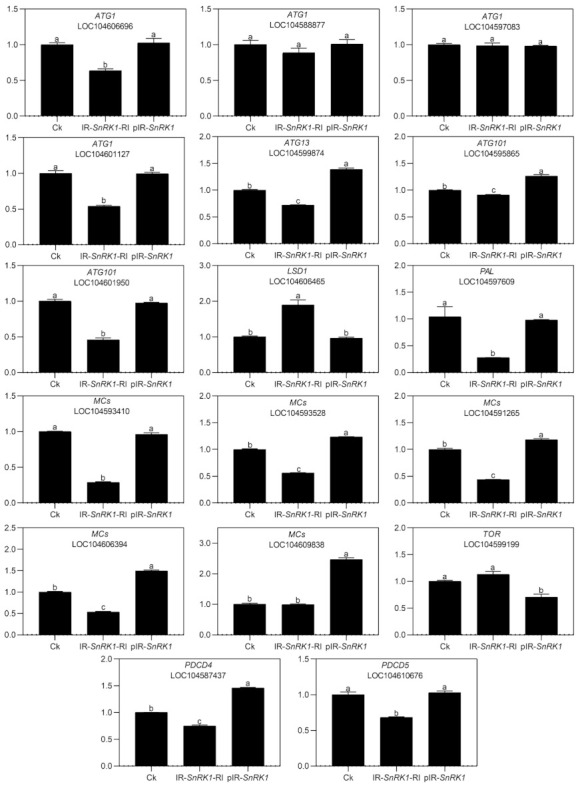
Expression analysis of autophagy- and programmed cell death (PCD)-related genes in the lotus. Two-month-old lotus seedlings were used for infection with IL-60-BS-derived systems. IR-*NnSnRK1*-RI, *NnSnRK1*-silenced lotus; pIR-*NnSnRK1*, *NnSnRK1*-overexpressed lotus. After 14 days of infection, the gene expression level was analyzed using quantitative real-time reverse transcriptase-polymerase chain reaction (qRT-PCR). Values are the means ± SE of three independent experiments with at least three replicates for each. Bars marked with different letters are significantly different from each other at *p* < 0.05, according to Tukey’s multiple test.

To explore the role of NnSnRK1 in inducing lotus flower bud abortion, we first confirmed its effectiveness by silencing the *NnSnRK1* gene (IR-*NnSnRK1*-RI) with the IL-60-BS-derived systems [25] in the lotus (Figure 6). This resulted in a significant reduction in the *NnSnRK1* gene expression level (Figure 6). The silenced lotus showed stronger flowering ability, with its flower:leaf ratio increased by 40% (Figure 6). This indicated that fewer flower buds were aborted in the *NnSnRK1*-silenced seedlings, probably an outcome of lower PCD in the flower bud cells. In both plants and animals, SnRK1 activates autophagy in response to nutrient deficiency or energy depletion [26]. Experimental evidence demonstrates that autophagy is an initiator of PCD [27] and necessary for developmental PCD in plants [28,29]. During lotus flower bud aborting, PCD also occurred (Figure 2), accompanied by the activation of NnSnRK1, which highlights the importance of NnSnRK1 in regulating PCD-induced flower bud abortion. To confirm this, we examined the expression of several key genes involved in autophagy execution in SnRK1-regulated signaling pathways. Many autophagy-related (ATG) proteins, including the direct target of SnRK1 (ATG1) and necessary components (ATG13/ATG11/ATG101), have been recently reported involved in autophagy [30]. As expected, most of them increased significantly in the aborting lotus flower buds (Figure 5). Upon starvation, repression of (TOR) signaling is another important way for SnRK1 to drive autophagy and induce growth repression [31]. In this work, we found that all identified *TOR* genes only slightly decreased in the aborting flower buds (Figure 5). This indicates that *ATG* genes might be the key target genes of SnRK1 in inducing autophagy, which in turn activates PCD in lotus flower buds. We further examined the expression of autophagy and PCD-related genes in IR-*NnSnRK1*-RI and pIR-*NnSnRK1* lotus seedlings. Results showed that positive regulator genes of autophagy (*ATG1*, *ATG11*, *ATG13,* and *ATG101*) and PCD (*PDCD4*, *PDCD5*, *PAL,* and *MCs*) were differentially downregulated in *NnSnRK1*-silencing seedlings (Figure 7). Meanwhile, the negative regulator gene of PCD (*LSD1*) was significantly upregulated in IR-*NnSnRK1*-RI seedlings (Figure 7). We also found that the expression of *TOR* showed few changes in IR-*NnSnRK1*-RI seedlings (Figure 7). Moreover, consistent with the flowering phenotype, overexpression of SnRK1 showed fewer changes in these genes (Figure 7). Together, we speculated that the SnRK1-ATG1s-mediated autophagy pathway might lead to PCD in lotus flower buds.

To our knowledge, this might be the first report on the role of SnRK1 in regulating flower bud abortion, although it was known as a negative regulator in the vegetative-reproductive transition [32]. Notably, overexpression of the *NnSnRK1* gene (pIR-*NnSnRK1*) has little or no effect on its flower:leaf ratio, even though higher levels of *NnSnRK1* mRNA were observed (Figure 6). This suggested that NnSnRK1 is necessary but might not be sufficient for inducing lotus flower bud abortion. Despite the considerable progress in understanding the SnRK1 signaling pathway over the last years, its components in regulating flowering remain to be further investigated.

### 2.6. NnSnRK1 Was a Negative Flowering Regulator in ABA Signaling

**Figure 8:**
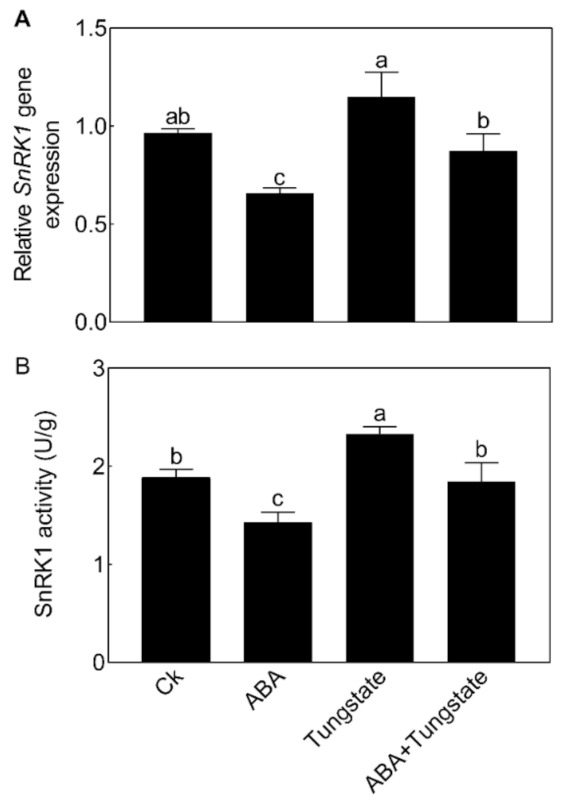
Effects of abscisic acid (ABA) and tungstate treatments on the lotus. Two-month-old lotus seedlings were treated with 80 μM ABA and 4.5 mM tungstate. After 9 days of treatment, the expression level (**A**) and activity (**B**) of NnSnRK1 in the flower buds were analyzed. Values are the means ± SE of three independent experiments with at least three replicates for each. Bars marked with different letters are significantly different from each other at *p* < 0.05, according to Tukey’s multiple test.

**Figure 9:**
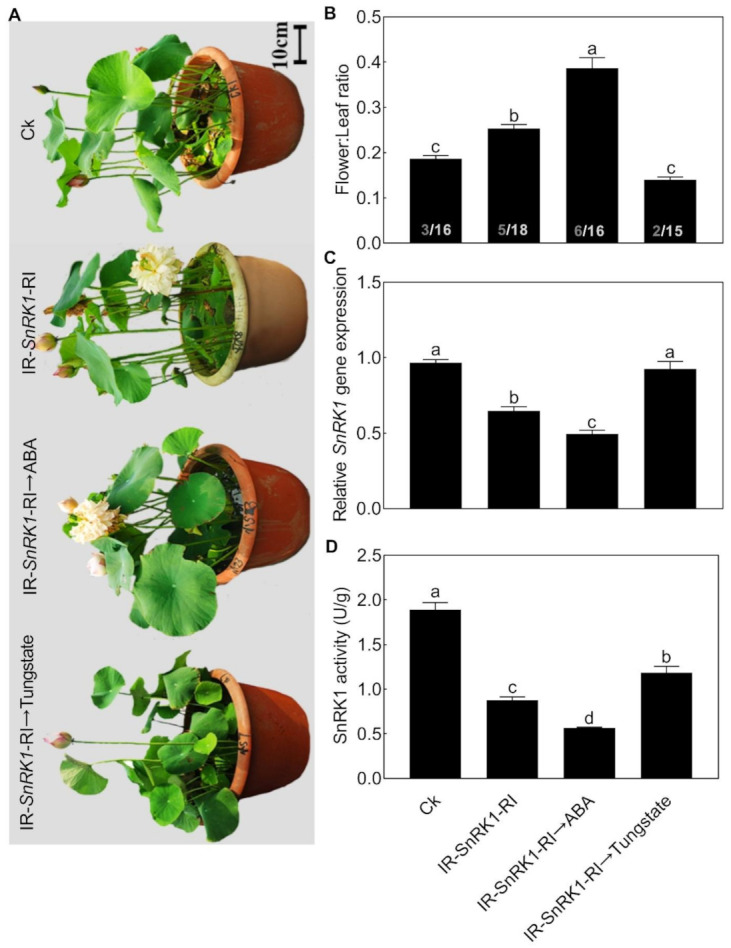
Effects of abscisic acid (ABA) and tungstate on *NnSnRK1*-silenced (IR-*NnSnRK1*-RI) and -overexpressed (pIR-*NnSnRK1*) lotus. Two-month-old lotus seedlings were used for infection with IL-60-BS-derived systems. IR-*NnSnRK1*-RI, *NnSnRK1*-silenced lotus; pIR-*NnSnRK1*, *NnSnRK1*-overexpressed lotus. After being infected for 14 days, seedlings were treated with ABA (80 μM) or tungstate (4.5 mM) for another 9 days. Then, the representative picture was taken (**A**) and the flower:leaf ratio (**B**), gene expression level (**C**), and activity of NnSnRK1 (**D**) were analyzed. *NnSnRK1*-silenced and -overexpressed lotus seedlings were generated with the IL-60-BS-derived systems. The numbers on the bottom of each column represent the corresponding number of flowers and leaves of the flower:leaf ratio. Values are the means ± SE of three independent experiments with at least three replicates for each. Bars marked with different letters are significantly different from each other at *p* < 0.05, according to Tukey’s multiple test.

We then further analyzed the relationship between NnSnRK1 and ABA. When seedlings were treated with ABA, the expression level (Figure 8A) and protein kinase activity (Figure 8B) of NnSnRK1 significantly decreased. The result was consistent with previous work in wheat and rice, which showed that ABA treatment can result in a drastic decline in SnRK1 [9,33]. The phenotype of *NnSnRK1*-silenced seedlings could also be enhanced by ABA treatment (Figure 9), with its flower:leaf ratio increased by 52%. Tungstate treatment caused the flower:leaf ratio of *NnSnRK1*-silenced seedlings to return to the ratio similar to the control (Figure 9). This might be explained by their activating or repressing effects on the expression level and activity (Figure 9) of NnSnRK1. In plant vegetative growth and stress response, however, ABA was reported as an activator of SnRK1 through inhibition of type 2C protein phosphatases [34,35]. Here, we revealed an additional unknown regulatory pathway of ABA/SnRK1 in controlling flower bud abortion, finding that NnSnRK1 is a negative flowering regulator in the ABA-mediated signaling pathway of flowering time control. We concluded that SnRK1 might act as an important developmental switch between vegetative/stress responses and reproductive development. It needs to be noted that NnSnRK1 is necessary, but might not be sufficient, for inducing lotus flower bud abortion, indicating that other components in this pathway remain to be identified. The above signaling pathway could help the lotus to terminate the flowering processes with low reproductive success, thereby ensuring that resources are optimally used and redirected in support of survival.

## 3. Materials and Methods

### 3.1. Plant Material and Reagents

*Nelumbo nucifera* cultivar ‘Xiaohongju’ was used for the different analysis. Uniform rhizomes of “Xiaohongju” were planted in plastic pots (48 cm in diameter) filled with soil. Lotus seedlings were grown in a greenhouse under natural light conditions from April to August (Nanjing, China) with a temperature range of 13–35 °C. Different treatments or observations were started on June. ABA ((+)-abscisic acid, purity ≥ 95%) and tungstate (Na_2_WO_4_, AR) are from the Yuanye biology company. Tungstate is a kind of inhibitor of ABA [35]. All experiments were performed in three independent experiments with at least three replicates.

### 3.2. Histochemical Staining

Evans blue staining was used to indicate the membrane damage of cell death [36]. We examined the production of superoxide anion (O_2_-) and hydrogen peroxide (H_2_O_2_) in situ by NBT [37] and 3, 3′-diaminobenzidine (DAB) staining, respectively [38].

### 3.3. TUNEL Analysis

The 2- to 3-cm-long excised flower buds were immediately fixed in 5% Formaldehyde-acetic acid-ethanol Fixative (FAA) fixation for 24 h. Then the fixed samples were dehydrated through graded ethanol and processed for paraffin embedding via standard methods [39]. The paraffin sections were stained with diaminobenzidine (DAB), fluorescein isothiocyanate (FITC), and 4′,6-diamidino-2-phenylindole (DAPI) using the In Situ Cell Death Detection Kit POD (Roche). The stained sections were observed using fluorescence microscopy (Olympus).

### 3.4. Hormone Contents in Aborting and Normal Lotus Flower Buds

ABA, auxin (IAA), salicylic acid (SA), gibberellin acid (GAs), cytokinin (CTK), and 1-aminocyclopropane-1-carboxilic acid (ACC, ethylene synthesis precursor) from the well-developed and aborting flower buds were assayed according to previously described methods [40,41,42,43]. The sample extracts were analyzed using an LC-ESI-MS/MS system (HPLC, Shim-pack UFLC SHIMADZU CBM30A system; MS, Applied Biosystems 6500 Triple Quadrupole). 

### 3.5. RNA-seq Library Preparation and Sequencing

For RNA-seq, the normal and aborting flower buds (2–3 cm in length) were collected. Each plant material has three biological replicates. Total RNA was isolated using the CTAB method. The library preparation of normal (Ck) and aborting flower (Ab) buds, Illumina sequencing, and data analysis were performed by the BGISEQ-500 sequencing platform (BGI-Shenzhen, China), as previously described [44]. The lotus genome (http://www.ncbi.nlm.nih.gov/genome/?term=nelmbo+nucifera, accessed on 15 April 2020) is used for read mapping. Expression values were normalized in FPKM (fragments per kilobase of exon per million fragments mapped). Adjusted *p*-value (Q-value) ≤ 0.001 and log_2_fold change ≥ 2 were set as the threshold for significantly differential expression. Sequence data have been deposited with the GenBank data libraries under accession number PRJNA707244.3.6. Gene Regulation of the Lotus by the IL-60 System

We performed gene expression or silencing of the lotus via modified tomato yellow leaf curl virus (TYLCV)-based geminivirus vector system (IL-60-BS/IR), which is a non-transgenic universal vector system for gene expression and silencing [45,46]. NnSnRK1 overexpression vector (pIR-*NnSnRK1*) or silencing vector (IR-*NnSnRK1*-RI) was injected into the two-month-old lotus seedling leaves together with IL-60-1 plasmid at a ratio of 1:1 (800ng/100uL). The phenotypes, gene expression level, and kinase activity of NnSnRK1 were analyzed at 14 or 23 days after infection.

### 3.6. qRT-PCR and Kinase Activity Analysis of NnSnRK1 

The total RNA from a lotus was prepared using a Vazyme reagent kit according to the manufacturer’s procedure. Reverse transcription was carried out with the Vazyme reagent kit, and qRT-PCR analysis was performed using ChamQ SYBR qPCR Master MIX (Applied VAZYME) with the QuantStudio^TM^ Real-Time PCR system. The relative transcript abundance of genes was determined by ΔCT method using actin as the reference gene [47]. The specific primers are listed in Table S1. A plant Snf1-related protein kinase (*SnRK1*) enzyme-linked immunoassay kit (Jonln biology, Shanghai, China) was used for SnRK1 kinase activity analysis.

### 3.7. Statistical Analysis

All data are shown as mean ± standard error. Statistical significance was validated using one-way analysis of variance (ANOVA) and with Tukey’s multiple range test or the independent samples *t*-test, with *p* < 0.05 considered statistically significant.

## Data Availability

The data that support the findings of this study are available from the corresponding author upon reasonable request.

## Supplementary Materials

The following are available online at https://www.mdpi.com/article/10.3390/ijms22083932/s1.
