# Supplementary material for: Regulation of Flowering Timing by ABA-NnSnRK1 Signaling Pathway in Lotus
Source: Int J Mol Sci. 2021 Apr 10;22(8):3932. doi: 10.3390/ijms22083932 (PMC8069233; doi:10.3390/ijms22083932)
Supplement: Supplementary file 1 [file ijms-22-03932-s001.pdf]

International Journal of
Molecular Sciences

MDPI

Article

Regulation of flowering timing by ABA-NnSnRK1 signaling pathway in lotus

Jing Cao†, Qijiang Jin†,*, Jiaying Kuang, Yanjie Wang and Yingchun XU *

College of Horticulture, Nanjing Agricultural University, Nanjing 210095, China
* Correspondence: jqj@njau.edu.cn (J.Q.J.); xyc@njau.edu.cn (X.Y.C.)
† These authors contributed equally to this study

Supplementary material:

Table S1. Primers used in the study.

Primer name	Forward primer (5′ to 3′)	Reverse primer (5′ to 3′)
SnRK1 104598048	CAGCTGGGGCAAGGGATTTA	AACCATGGGTGCTTTCGGAT
ATG1 104606696	CTGTTTGGGAGTCTGTGGA	ATGTCCTTCTGTGCCTTCTT
ATG1 104588877	ATCCAGGCTGAAGGGTA	GATGAATGATGTGGTGGG
ATG1 104597083	CCTCCTCACCATCACTAAG	GCTCCAAAGCATCTCCC
ATG1 104601127	TGGGTGAGATGGACAGTGC	ATGTCCGAAGACGAAATAGA
ATG11 104612475	TAGAGTCGGTATCAGGTATTCAC	CGCACGGAAGTTTGTAGGC
ATG13 104586254	CATCGCCTTCCCTAAAA	CTCATCCCTCCCAGCATAG
ATG101 104595865	CTTCTCAAGCCTCAACCC	GCACTAAATAAACCCGCAAA
ATG101 104601950	AGCAGCCTTGGAAACATCT	TCTGATGAACTCGGGATTG
TOR 104599199	TCGGCGAGGGAGAAT	CCTTGTAGACGAGGATGG
LSD1 104606465	GTGGAGGGAATGGAAGGC	CGTTAGGAGGCGTAAATGGT
PDCD4 104587437	TTTCTGCGGGACTTATCACT	AACCGTTCTCCACCTCATT
PDCD5 104610676	GGTTTCGCTGCTTCTTCCA	CCTTTTCGCCTCTTCCTGT
PAL 104597609	AACGCAACTGCCATCC	TAACAAGCCCACATCCT
MCs 104593410	TTTATTAGGTGAGCGACAAG	CGGGAATAGGAGGTTGA
MCs 104593528	CGACATCGTGCTACTGACC	GCCCGCTGTAATGGAAG
MCs 104591265	AGAAGACAGGGAAGAACGAA	AACTCCAGAGCCCGATTT
MCs 104606394	CTTACCAACCTCCGCTACCG	GCCCTTGAGTTCGTGCC
MCs 104609838	CAATGACGCCAAGTGC	GTGAACCGTGACCAGAATAG
Actin 104593066	GCGTTCTGCCGTCTTCTAAA	CCCTCTTGGATTGTGCCTC

Note: Numbers after gene names represent NCBI accession IDs.
